# The association between autistic traits and trajectories of anxiety in middle-aged and older adults: an 8-year growth mixture model analysis

**DOI:** 10.1038/s44220-026-00654-0

**Published:** 2026-06-01

**Authors:** Aphrodite Eshetu, Saloni Ghai, Anne Corbett, Clive Ballard, Adam Hampshire, Elizabeth O’Nions, William Mandy, Joshua Stott, Amber John, Gavin R. Stewart

**Affiliations:** 1 Research Department of Clinical, Educational and Health Psychology, University College London, London, UK; 2 Medical School, University of Exeter, Exeter, UK; 3 Department of Neuroimaging, King’s College London, London, UK; 4 Bradford Institute for Health Research, Bradford Teaching Hospitals NHS Foundation Trust, Bradford, UK; 5 Department of Psychology, Institute of Population Health, University of Liverpool, Liverpool, UK; 6 Social Genetic and Developmental Psychiatry Centre, King’s College London, London, UK

**Keywords:** Autism spectrum disorders, Anxiety

## Abstract

Anxiety is a highly prevalent mental health condition, and it is particularly common in autistic populations. However, little is known about its course beyond midlife in autistic populations owing to limited longitudinal research. Here we analyzed data from 5,270 adults aged 50–91 years (median 62 years; 75% female) in the PROTECT study. Participants completed measures of autistic traits (the Autistic Spectrum Traits Questionnaire) and current anxiety symptoms (the Generalized Anxiety Disorder-7 questionnaire). In total, 66 participants (~1.3%; 72.3% female) had high autistic traits (AST group), while 3,874 (~73.5%; 77.8% female) reported none (comparison adults). Growth mixture modeling identified anxiety symptom trajectories over an 8-year annual follow-up period. Associations between AST group and trajectory membership were examined using multinomial logistic regression. Growth mixture modeling revealed three class trajectories: two representing persistently minimal anxiety symptoms (class 1, ‘lower range minimal’, 85.6% of sample; class 2, ‘upper-range minimal’, 12.4% of sample) and one showing rising anxiety from ‘mild to clinical’ levels (class 3, 2% of sample). Regressions showed that AST participants were more likely than comparison adults to follow the mild-to-clinical trajectory (relative risk 4.41, 95% confidence interval 1.70–11.44). Elevated autistic traits are associated with an increased risk of worsening anxiety with age, highlighting the need for tailored support.

## Abstract (web summary)

Eshetu and collaborators investigated anxiety comorbidity in later life among adults with elevated autistic traits, examining how anxiety symptoms unfold with aging in a large UK community sample.

## Main

Autism spectrum disorders, henceforth ‘autism’, are a set of early onset neurodevelopmental conditions characterized by differences in sociocommunicative abilities and rigid and repetitive behaviors and interests^1^. Autism is a lifelong condition and has a global prevalence of approximately 1%^2^. While not a part of diagnostic criteria, autistic people across the lifespan often have co-occurring mental health difficulties^3–7^, with systematic reviews finding prevalence rates of 20–23% (current) and 42% (lifetime) for anxiety disorders^8,9^. Notably, anxiety has been identified as one of the top two priorities in autism research by autistic people themselves^10^. While anxiety has been well studied in younger autistic populations^3,4^, only a small number of studies have specifically examined the rates of anxiety diagnoses^5–7,11,12^ and prevalence of anxiety symptoms^6,7,13–17^ in middle-aged and older people on the autism spectrum. As such, our current understanding of the burden and course of anxiety in autistic populations in midlife and older age is limited.

At present, only one follow-up study has examined mental health changes in middle aged and older autistic adults^18^. Over an average follow-up period of 2.4 years, the study found no significant change in anxiety symptoms. However, the short follow-up window limits conclusions about longer-term trajectories associated with aging. Cross-sectional studies provide mixed evidence regarding the rates of anxiety across different age groups in autism. There has been evidence to suggest lower rates of clinical anxiety in older autistic populations compared to younger autistic age groups^19^, while other studies have found the opposite pattern^20^. Still other studies identify complex age associations, such as cubic trends where symptoms decrease in younger adulthood and resurge in midlife^21^ or no significant differences across the lifespan altogether^22^. These inconsistencies, coupled with the limitations of cross-sectional designs and potential cohort effects, underscore the need for more longitudinal research to clarify developmental patterns in anxiety across the adult lifespan in autism.

There are two main issues that surface when trying to address this gap. The first is the issue of underdiagnosis among those who are middle-aged and older. Estimates suggest that nine out of every ten autistic adults in this age range are undiagnosed^23,24^, which is probably the result of the changes to the diagnostic criteria and conceptualization of autism in the past 50 years^25,26^. This impedes recruitment of middle-aged and older autistic people with a diagnosis to research studies. A trait-based approach offers a valuable alternative to diagnosis-based recruitment, particularly in midlife and older populations where autism is frequently underrecognized or misdiagnosed^27^. This approach allows researchers to identify people with elevated autistic traits and characteristics who may not have received a formal diagnosis, thus capturing a broader and potentially more representative sample of autistic adults.

The second consideration is the heterogeneity associated with the longitudinal course of anxiety between individuals^28^, which standard repeated-measure analyses cannot capture. This has been addressed in general population studies using longitudinal forms of latent class analysis and growth mixture modeling (GMM) methods. These statistical procedures allow the modeling of individual trajectories of a given outcome and the subsequent identification of previously unobserved subpopulations within a heterogeneous sample^29^.

The aim of the current study is to use GMM to identify distinct anxiety trajectories in a large, longitudinal cohort of middle-aged and older adults and examine whether endorsement of sociocommunicative autistic traits is associated with trajectory class membership. These results could provide insight into the experience of aging with autism and improve understanding of the mental health support needs of this understudied population.

## Results

### Demographics and lifetime psychiatric diagnoses

All descriptive statistics for the overall sample and by group and are presented in Table 1. The majority of the sample reported their biological sex as female (75%), and there was an underrepresentation of Black, Asian and Minority Ethnic (BAME) people (1.3%) compared with the general population. The median age at baseline was 62 years. While age, gender ratio and ethnicity did not differ between the autism spectrum trait (AST) and comparison adults (COA) groups, higher baseline depression scores (Patient Health Questionnaire-9, PHQ-9) were observed in the AST group. However, prevalence rates of lifetime diagnoses of major depressive disorder and generalized anxiety disorder did not differ between groups. By contrast, larger proportions of the AST group reported having lifetime diagnoses of social anxiety, panic disorder and obsessive–compulsive disorder (OCD). Furthermore, no participant in the overall sample had an autism diagnosis.

**Table 1:** Descriptive statistics for total sample and by group at baseline

		Total sample	COA	AST	Group difference
Age (years)	Median (IQR)	62 (57–66)	61 (57–66)	60 (55–66)	*z* = 1.335, *P* = 0.182
	Range	50–91	50–91	50–79	
Sex	Male:female	1,315:3,955	861:3,013	18:48	*χ*^2^ = 0.954, *P* = 0.329
	%	25.0%:75.0%	22.2%:77.8%	27.3%:72.3%	
Ethnicity	White:BAME	5,201:69	3,825:49	66:0	*χ*^2^ = 0.845, *P* = 0.358
	%	98.7%:1.3%	98.7%:1.3%	100%:0%	
Depression score at baseline, T0	Median (IQR)	2 (0–3)	1 (0–3)	5 (2–9)	*z* = −7.74, *P* = 0.001***
	Proportion above cutoff	191 (3.6%)	86 (2.2%)	12 (18.2%)	*z* = 8.31, *P* < 0.001***
Major depressive disorder diagnosis		1,270 (70.8%)	811(20.9%)	32 (48.5%)	*z* = 5.42, *P* < 0.001***
Generalized anxiety disorder diagnosis		719 (13.6%)	471 (12.2%)	21 (31.8%)	*z* = 4.85, *P* < 0.001***
Social anxiety diagnosis		50 (0.9%)	22 (0.57%)	9 (13.6%)	*z* = −7.05, *P* < 0.001***
Panic disorder diagnosis		269 (5.1%)	162 (4.2%)	11 (16.7%)	*z* = 3.19, *P* < 0.001***
OCD diagnosis		12 (0.2%)	4 (0.1%)	1 (1.5%)	*z* = 3.21, *P* < 0.001***

IQR, interquartile range. ***P < 0.001.

Using a subsample of the full PROTECT cohort with sufficient longitudinal data for GMM analysis (that is, at least baseline plus two annual follow-up assessments), a total sample of 5,270 participants (*n* = 3,955 female; 75.0%) was included in the analyses, of whom 66 (1.25%) met cutoff criteria for the high AST group (see ‘Measures’ section in the Methods for inclusion criteria). Participants who were found to endorse no traits using this measure formed the COA group (*n* = 3,874, 73.51%). Participants with missing scores (*n* = 237, 4.50%) and those who endorsed some traits but fell below the cutoff (*n* = 1,093, 20.74%) were also included in the GMM to maximize statistical power and trajectory identification but were excluded from further analyses (Table 2).

**Table 2:** Proportion of AST and COA group membership across latent classes

	Class 1: lower-range minimal	Class 2: upper-range minimal	Class 3: mild-to-clinical range
AST (*n*, %)	38 (57.58)	20 (30.30)	8 (12.12)
COA (*n*, %)	3,417 (88.20)	409 (10.56)	48 (1.24)

### Model selection

Comparison of one-class growth models revealed that a quadratic GMM provided better fit than a linear model (Supplementary Tables 1–4). However, it was found that quadratic models with three or more classes were converging on improper solutions, as the estimated correlation between the linear and quadratic slopes was almost perfectly negative in both cases. The variance of the quadratic term was also very small, which made the models susceptible to instability during estimation. To address this, the variance of the quadratic term was fixed to be 0.

### Trajectories of anxiety symptoms change for total sample

**Fig. 1:**
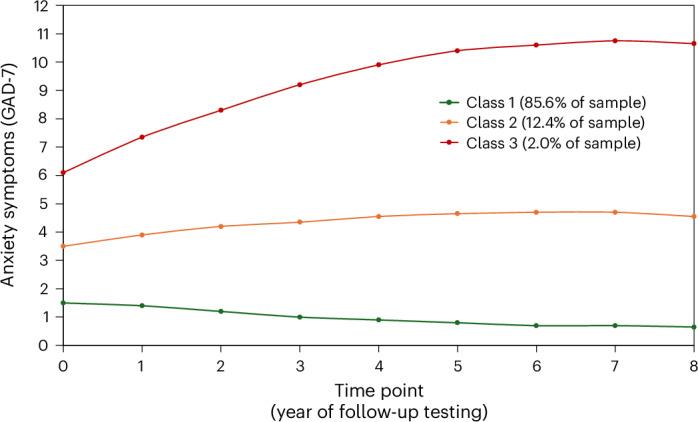
Anxiety trajectory classes from GMM. A three class model, with ‘class 1’ representing lower-range minimal anxiety, ‘class 2’ representing upper-range minimal anxiety and ‘class 3’ representing mild-to-clinical anxiety.

GMM analysis was conducted on the entire sample (*n* = 5,270; AST 66, COA 3,874, missing AST score 1,330). Overall, a three-class model was selected as the final model (see Supplementary Tables 1–4 for model fit statistics and Supplementary Table 5 for class-stratified demographics). While the Akaike information criterion (AIC), Bayesian information criterion (BIC) and sample-size-adjusted BIC (ABIC) values were lower for the three-class model, the adjusted Lo–Mendel–Rubin (LMR) and adjusted likelihood ratio test (ALRT) indices were no longer significant in the three-class model. These metrics can be sensitive to sample size and may not detect small but meaningful classes. As such, we prioritized the three-class model based on AIC, BIC, ABIC and interpretability, and because it captured a distinct subgroup of 56 people (1.06% of the full sample) with clinically significant anxiety trajectories, a trajectory not identified in the two-class model. For the same reason, models with more than three classes were not considered. As estimated prevalence rates of anxiety disorders in older adult community samples range from 1.2% to 15%^30^, we opted to retain this class. The identified trajectories are shown in Fig. 1 and were described on the basis of standard Generalized Anxiety Disorder-7 (GAD-7) symptom thresholds^31^: ‘class 1’, lower-range minimal anxiety; ‘class 2’; upper-range minimal anxiety; and ‘class 3’, mild-to-clinical anxiety.

Class 1 was the largest trajectory class, comprising 85.6% of the sample. People in this class were characterized by estimated GAD-7 scores ranging from 0.5 to 1.5 over the eight years, remaining at the lower end of the ‘minimal anxiety’ range (scores 0–4)^32^. Membership in this class was more frequently observed in the COA group (88.2%), compared with the AST group (57.58%). Class 2 consisted of 12.4% of the total sample and showed anxiety scores that persisted in the upper end of what is labeled ‘minimal anxiety’. The reverse pattern to class 1 is seen here, in that a greater proportion of the AST group were represented by this class (30.3%) compared with the COA group (19.56%). Class 3, the smallest of the three trajectories identified, 2%, was the only class to show a notable change in anxiety, with scores gradually rising from mild to clinical levels over time. Again, it was more common for AST participants to belong to this class (12.12%) than the COA participants (1.24%).

Associations between sociocommunicative autistic traits and class membership were investigated using unadjusted (Table 3) and adjusted (Tables 4 and 5) multinomial logistic regression models, using class 1 (lower-range minimal anxiety) as the reference group. In the unadjusted model, AST participants had 4.40 times the relative risk (RR) of belonging to class 2 (versus class 1), and 15.00 times to the RR of belonging to class 3 (versus class 1) compared with COA participants. This effect remained consistent when adjusting for sex and age (Table 4). While the effect size was attenuated when adjusting for baseline depression (PHQ-9), AST participants were still at significantly greater likelihood of belonging to class 2 and more so class 3 than COA participants.

**Table 3:** Association between autistic traits and anxiety trajectory class relative to class 1

Baseline predictor	Class 2 RR (95% confidence interval), *P* value	Class 3 RR (95% confidence interval), *P* value
AST score	4.40 (2.53–7.63), *P* < 0.001	15.00 (6.22–33.82), *P* < 0.001

**Table 4:** Association between autistic traits and anxiety trajectory class controlling for shown covariates relative to class 1

Baseline predictor	Class 2 RR (95% confidence interval), *P* value	Class 3 RR (95% confidence interval), *P* value
AST score	4.46 (2.56–7.75), *P* < 0.001	14.60 (6.40–33.30), *P* < 0.001
Sex: female	1.39 (1.07–1.82), *P* = 0.015	1.25 (0.62–2.51), *P* = 0.610
Age	0.98 (0.97–1.00), *P* = 0.028	0.93 (0.89–0.97), *P* < 0.001

**Table 5:** Association between autistic traits and anxiety trajectory class controlling for shown covariates relative to class 1

Baseline predictor	Class 2 RR (95% confidence interval), *P* value	Class 3 RR (95% confidence interval), *P* value
AST score	2.26 (1.23–4.15), *P* = 0.008	4.41 (1.70–11.44), *P* = 0.002
Sex: female	1.32 (1.01–1.73), *P* = 0.045	1.29 (0.61–2.72), *P* = 0.501
Age	0.99 (0.98–1.01), *P* = 0.345	0.96 (0.91–1.00), *P* = 0.052
Depression	1.25 (1.21–1.29), *P* < 0.001	1.39 (1.31–1.47), *P* < 0.001

## Discussion

This study provides longitudinal evidence that the heightened risk of anxiety faced by those with elevated autistic traits persists into midlife and older age. In our sample of over 5,200 middle-aged and older adults, we identified three trajectories of anxiety symptoms over an 8-year period. The two largest classes (class 1: 85.6% of sample, class 2: 12.4% of sample) were characterized by anxiety scores within the minimal symptom range (0–5) of the commonly used anxiety symptom measure, the GAD-7. Although these two classes were identified as having distinct probabilities of anxiety, due to their low scores, they are unlikely to be differ greatly in terms of clinical presentation. The third and smallest class (class 3: 2% of sample) showed a curved anxiety trajectory where scores gradually increased from mild to clinical anxiety over the eight annual time points studied. Although this class comprised only 2% of the sample, given the large total sample size, robust detection of distinct, smaller classes was feasible. These findings are consistent with previous GMM studies in general population samples that have also identified small class sizes in mental health trajectories^33,34^.

When considering the distribution of the high autistic trait (AST) and low autistic trait comparison (COA) groups, we found these to vary in proportion across classes. A greater proportion of AST participants were classified into class 3 (mild-to-clinical trajectory; AST 12.12%, COA 1.24%) and class 2 (upper-range minimal anxiety trajectory; AST 30.30%, COA 10.56%), whereas most COA participants were classified into class 1 (lower-range minimal anxiety; AST 57.58%, COA 88.20%). Although members of the AST group were found across all three classes, multinomial logistic regression showed that, relative to class 1, AST participants were upward of 14 times more likely to belong to class 3 than COA participants, an effect that remained after adjusting for depression scores, although attenuated. This suggests that some middle-aged and older people with high autistic traits are more susceptible to high and persistent anxiety than their low-trait peers.

The finding that the AST group was overrepresented in class 3 aligns with the findings from both cross-sectional studies^5,7^ and the only other longitudinal follow-up study of mental health in ageing in relation to autism^18^, which similarly found that high levels of anxiety were sustained over time. However, our use of GMM revealed a steady increase from subclinical to clinical GAD-7 scores (≥10) observed in class 3, highlighting the need for early intervention strategies for anxiety. Indeed, previous research has demonstrated that treatment of mild psychological disorders can significantly reduce the proportion of future more severe cases^35^. Adapted forms of low-intensity cognitive behavioral therapy have proved successful for mild-to-moderate depression in autistic adults^36^, suggesting the potential for similar approaches in addressing subclinical anxiety.

There are several possible reasons why some middle-aged and older adults with elevated autistic traits might face an increased risk for greater anxiety. Studies have found that autistic traits are associated with an increased risk of experiencing loneliness and isolation^13^, trauma across the lifespan, and the impact of severe trauma on PTSD symptoms^15,37–39^. Autistic people are also more likely to experience barriers in health care services, so they may be less likely to receive appropriate treatment for anxiety^40–43^. A lifetime of these adverse experiences is likely to contribute to discrepancies in mental health difficulties faced by autistic and non-autistic adults beyond midlife.

It will be important to consider how the barriers to receiving mental health care for autistic people (for example, communication difficulties and limited specialized knowledge of mental health in aging with autism) will be compounded with other upper-age barriers (for example, stigma around mental illness that prevents help-seeking)^44^. Autism and mental health research must therefore consider this intersectionality going forward. In addition, it is important to consider the specificity of these findings; for example, are these experiences and patterns unique to autistic populations, or are they also observed in other neurodivergent groups (for example, people with attention deficit hyperactivity disorder) or in individuals with long-term disabilities and health conditions? Cross-group comparative studies would facilitate this type of exploration and should be considered in future research.

### Limitations and strengths

It will also be important to consider the present findings in light of the following limitations. First, the current study was not preregistered. Second, as PROTECT is an ongoing study, baseline is tied to the point of entry for each participant. Consequently, these trajectories cannot be localized to a certain age range. Also, this study excluded participants who took part in fewer than three time points, the minimum requirement for estimation of GMMs^45^. However, people with psychiatric disorders often show higher attrition^46^ and therefore may not be fully represented in this study. Diversity and representation were also limited by the demographic characteristics of the PROTECT sample, which is predominately made up of white, female, well-educated participants, with no BAME people in the AST group at all. However, this issue is not unique to the PROTECT cohort and is common in both aging and autism research fields^47,48^. It is also worth noting that anxiety is often more common in women than men^49^. Our class 2 and 3 groupings did have significantly more women than men in them, which aligns with these anxiety sex/gender differences observed in the general population. As such, given the overrepresentation of individuals of white ethnicity and females in our sample, future research should seek to replicate these findings in ethnically diverse and sex- and gender-balanced samples. Also, consideration should specifically be given to middle-aged and older BAME autistic people as studies like ours may not be representative of their experiences. Furthermore, a final point to consider is that the AST group was relatively small, particularly in the mild-to-clinical trajectory; this may reduce statistical precision, and as such, the results should be interpreted in this context. Replication of these findings in larger samples of diagnosed autistic people would be valuable.

However, there are notable strengths in the current study. Although the AST sample was small and derived from a brief screener (which focuses on sociocommunicative difficulties but has good sensitivity and specificity for identifying autistic individuals), it represented only 1.3% of the overall sample. Although we cannot confirm whether these identified high autistic trait participants would meet diagnostic criteria for autism, the overall group proportion identified aligns with epidemiological estimates of autism within the general population (~1%)^2,23^. In addition, the overall sample of 5,270 allows robust GMM estimation. Furthermore, the number of classes included in the final GMM was driven by the data (rather than an a priori decision). The iterative process used in GMM supported the three-class model. However, this solution should be interpreted with some caution, given mixed fit indices and the relatively small size of class 3. The high entropy suggests good classification accuracy for the three-class model. We note that that this autistic-trait-based work should be replicated using more diverse samples, including those with a diagnosis of autism, intellectual disabilities and different cultural and ethnic contexts. This will allow a more comprehensive understanding of the risk of anxiety in this population.

## Conclusion

Three distinct anxiety trajectories were identified in this large general population sample of middle-aged and older adults. Participants with elevated sociocommunicative autistic traits were found to be at a greater risk of experiencing higher anxiety symptoms over time. Given the detrimental consequences of chronic anxiety for physical and mental health, these findings highlight the need for increased, tailored support for adults on the autism spectrum. Researchers, clinicians and health policymakers should aim to collaborate with autistic people where possible, as their lived experience affords vital insight into their support needs during the transition into midlife and older age.

## Methods

### Study design and participants

The present study uses 8 years of annual longitudinal data from the PROTECT study (www.protectstudy.org.uk). PROTECT is an ongoing, online research study with annual follow-ups based in the UK that aims to examine aging and health. The PROTECT study uses a wide range of recruitment methods, including public and national press and radio, as well as established aging research networks. Potential participants are directed to register online via the PROTECT platform and are required to review an information sheet and to provide informed consent to take part. Participants are required to meet the following inclusion criteria for the PROTECT study: be at least 40 years of age (due to the focus on mid-to-late life), have a working proficiency of the English language, be a resident of the UK and have access to a device with Internet access. The only two exclusion criteria for the PROTECT study are: having a dementia diagnosis at baseline assessment and being unable to consent.

The PROTECT study was piloted in 2014 and publicly commenced in 2015. As an ongoing study, baseline is defined as each participant’s point of entry and is not study specific. Participants receive annual follow-up requests for participation via email. While the present study is an analysis of up to eight consecutive years of assessment, those who joined PROTECT in more recent years have fewer time points of annual follow-ups.

Ethical approval for the PROTECT study was obtained from the London Bridge National Research Ethics Committee (reference 13:/LO/1578).

### Measures

#### Demographic information and medical history

All participants who participated in the PROTECT study completed an online demographic information questionnaire at baseline, which collected data including participants’ age, sex, ethnicity and several lifetime psychiatric diagnoses (major depressive disorder, generalized anxiety disorder, panic disorder, social anxiety and OCD; Table 1).

#### Autistic traits

The present study used the bespoke PROTECT Autistic Traits measure, which is a five-item screener that assesses childhood and current sociocommunicative autistic traits^7^. This measure was completed at baseline. Using a yes/no format, the participant was asked if as a child they had “struggled compared to [their] peers (socially or at school) with: (1) knowing how to get along with other children; (2) understanding other kids’ jokes, sarcasm or deception.” Further questions asked if the participant “currently find[s] it more difficult than other people to: (1) make and keep friends; (2) understand other people’s perspectives; (3) recognize if someone means something different from what they are saying.” To meet the cutoff for the AST group, participants needed to endorse both childhood traits and at least two of the three current traits. Those who did not endorse any past or present autistic traits formed the COA group.

The PROTECT Autistic Traits measure has been found to have high sensitivity (82%) and specificity (96%) for identifying people with an autism diagnosis, good convergent validity with other widely used autistic trait measures (that is, the AQ-10^50^ and RAADS-14^51^) and very good internal consistency (Cronbach’s *α* = 0.82)^7^.

#### Self-report questionnaire measures

GAD-7^31^ is a widely used seven-item questionnaire that assesses symptoms of anxiety experienced in the past 2 weeks. Using the standard cutoff of ≥10, the GAD-7 has a sensitivity of 89% for identifying people with generalized anxiety disorder. Recent symptoms of major depressive disorder were measured using PHQ-9^52^, a nine-item measure, with a cutoff for clinical cases of ≥10. The GAD-7 and PHQ-9 were completed at baseline and at each annual follow-up entry.

### Plan of analysis

GMM was used to estimate trajectories of anxiety symptoms over the eight waves of testing (using data collected at baseline then at each subsequent annual follow-up entry). Anxiety (GAD-7) scores from all participants (that is, *n* = 5,270) were included in the estimation to maximize sample size. Linear and quadratic growth models were compared to determine which provided a better fit to the data. Models with increasing numbers of classes were estimated and compared.

As there were no prior hypotheses about the number of classes, quadratic GMMs with *K* classes were sequentially compared with *K* − 1 classes. Several relative fit information criteria are available for consideration when comparing GMMs. These include the AIC, the BIC, the ABIC, the entropy, the Vuong–Lo–Mendell–Rubin test, and adjusted LMR and ALRT tests. The proportion of the smallest group was also considered. All GMM analyses were conducted using Mplus 8.1^53^.

Multinomial logistic regression models unadjusted and adjusted for demographic characteristics and depression were run using Stata version 17^54^ to assess the association between sociocommunicative autistic traits and class membership.

### Missing data

Participants who had total GAD-7 scores for fewer than three time points (that is, baseline plus two annual follow-up entries) were excluded from GMM analysis, as this is the recommended minimum requirement for proper estimation of GMMs^45^. Full information maximum likelihood was used to handle missing data on GAD-7 scores.

### Reporting summary

Further information on research design is available in the Nature Portfolio Reporting Summary linked to this article.

## Supplementary information

Supplementary Information Supplementary Tables 1–5.

Reporting Summary

## Data Availability

Due to data access and sharing restrictions, the data used in this study are not publicly available. For further information about data access, please contact the corresponding authors.
