# Supplementary material for: The association between autistic traits and trajectories of anxiety in middle-aged and older adults: an 8-year growth mixture model analysis
Source: Nat Ment Health. 2026 Jun 1;4(6):971–7. doi: 10.1038/s44220-026-00654-0 (PMC13259919; doi:10.1038/s44220-026-00654-0)
Supplement: Supplementary file 1 — Supplementary Tables 1–5. [file 44220_2026_654_MOESM1_ESM.pdf]

nature mental health

Supplementary information

https://doi.org/10.1038/s44220-026-00654-0

The association between autistic traits and trajectories of anxiety in middle-aged and older adults: an 8-year growth mixture model analysis

In the format provided by the authors and unedited

The Association between Autistic Traits and Trajectories of Anxiety in Middle-Aged and Older Adults: An 8-year Growth Mixture Model Analysis.

Supplementary materials

Supplementary Table 1. Fit indices for the linear vs quadratic one-class model (*n* = 5,270)

Fit indices	One-class linear	One-class quadratic
Parameters	11	15
AIC	136628.902	136217.107
BIC	136701.169	136315.654
ABIC	136666.215	136267.989

Supplementary Table 2. Fit indices for one- to three- class quadratic growth mixture model specifications with quadratic term fixed to 0 (*n* = 5,270)

**Fit indices**	**One-class**	**Two-class**	**Three-class**
Parameters	15	19	**23**
AIC	189291.332	186908.848	**185718.902**
BIC	189389.878	187022.674	**185870.007**
ABIC	189342.213	186973.298	**185796.921**
Entropy	NA	0.959	**0.942**
Smallest group %	NA	7.247	**1.9**
LMR *p*-value	NA	0.0001	**0.1511**
ALRT *p*-value	NA	0.0001	**0.1579**

Supplementary Table 3. Proportion of AST and COA participants within each class

	**Lower-range minimal**	**Upper-range minimal**	**Mild-to-clinical**
AST (n, %)	38 (0.84)	20 (3.18)	8 (7.77)
COA (n, %)	3,417 (85.23)	409 (65.13)	48 (46.6)

Note: AST = autism spectrum trait; COA = control older adults.

Supplementary Table 4. Class description of the quadratic three-class growth

	**Lower-range minimal**	**Upper-range minimal**	**Mild-to-clinical**
Average latent class probability (%)	96.5	98.2	91.1
Count (proportion of total sample%)	4,510 (86.12)	655 (12.4)	103(2.0)

Supplementary Table 5. Demographic characteristics stratified by class groups.

		Class 1 (n=4,539)		Class 2 (n=628)		Class 3 (n=103)		Group differences
**Age**	*Mean (SD)*	61.97	(6.69)	61.21	(6.75)	59.25	(6.63)	No statistical difference
	Min-Max	50-80		50-91		50-78		
**Sex**	*Male : Female*	1175 : 3364		122 : 506		18 : 85		Class 3 = Class 2 > Class 1
	%	25.9% : 74.1%		19.4% : 80.6%		17.5% : 82.5%		
**Anxiety**	*Mean (SD)*	1.04	(1.89)	3.44	(3.07)	6.38	(4.88)	Class 3 > Class 2 > Class 1
	Min-Max	0-19		0-19		0-19		
**Depression**	*Mean (SD)*	2.05	(2.53)	4.21	(3.47)	7.33	(5.68)	Class 3 > Class 2 > Class 1
	Min-Max	0-23		0-25		0-24		
**Autistic Traits**	*Mean (SD)*	0.35	(0.79)	0.61	(1.06)	1.22	(1.50)	Class 3 > Class 2 > Class 1
	Min-Max	0-5		0-5		0-5		

Note: Classes are formed based on anxiety symptom scores. Total class numbers include those with missing autistic trait scores.
