# Supplementary material for: The association between autistic traits and trajectories of anxiety in middle-aged and older adults: an 8-year growth mixture model analysis
Source: Nat Ment Health. 2026 Jun 1;4(6):971–7. doi: 10.1038/s44220-026-00654-0 (PMC13259919; doi:10.1038/s44220-026-00654-0)
Supplement: Supplementary file 2 — Reporting Summary [file 44220_2026_654_MOESM2_ESM.pdf]

nature portfolio

Corresponding author(s): Gavin Stewart
Joshua Stott

Last updated by author(s): 27/02/2026

Reporting Summary

Nature Portfolio wishes to improve the reproducibility of the work that we publish. This form provides structure for consistency and transparency in reporting. For further information on Nature Portfolio policies, see our Editorial Policies and the Editorial Policy Checklist.

Statistics

For all statistical analyses, confirm that the following items are present in the figure legend, table legend, main text, or Methods section.

n/a	Confirmed	
☐	☒	The exact sample size (n) for each experimental group/condition, given as a discrete number and unit of measurement
☐	☒	A statement on whether measurements were taken from distinct samples or whether the same sample was measured repeatedly
☐	☒	The statistical test(s) used AND whether they are one- or two-sided<br>*Only common tests should be described solely by name; describe more complex techniques in the Methods section.*
☐	☒	A description of all covariates tested
☐	☒	A description of any assumptions or corrections, such as tests of normality and adjustment for multiple comparisons
☐	☒	A full description of the statistical parameters including central tendency (e.g. means) or other basic estimates (e.g. regression coefficient) AND variation (e.g. standard deviation) or associated estimates of uncertainty (e.g. confidence intervals)
☐	☒	For null hypothesis testing, the test statistic (e.g. F, t, r) with confidence intervals, effect sizes, degrees of freedom and P value noted<br>*Give P values as exact values whenever suitable.*
☒	☐	For Bayesian analysis, information on the choice of priors and Markov chain Monte Carlo settings
☐	☒	For hierarchical and complex designs, identification of the appropriate level for tests and full reporting of outcomes
☐	☒	Estimates of effect sizes (e.g. Cohen's d, Pearson's r), indicating how they were calculated

Our web collection on statistics for biologists contains articles on many of the points above.

Software and code

Policy information about availability of computer code

Data collection	The present study uses eight-years of annual longitudinal data from the PROTECT study (www.protectstudy.org.uk). The PROTECT study was piloted in 2014 and publicly commenced in 2015. As an ongoing study, baseline is tied to the point of entry for each participant and not study specific. Participants receive annual follow-up requests for participation via email. While the present study is an analysis of up to eight consecutive years of assessment, those who joined PROTECT in more recent years have fewer timepoints of annual follow-ups.
Data analysis	Growth mixture modelling was used to estimate trajectories of anxiety symptoms over the eight waves of testing (using data collected at baseline then at each subsequent annual follow-up entry). Linear and quadratic growth models were compared to determine which provided better fit to the data. Models with increasing numbers of classes were estimated and compared. All GMM analyses were conducted using Mplus 8.1.34 Multinomial logistic regression models unadjusted and adjusted for demographic characteristics and depression were run using Stata version 1735 to assess the association between socio-communicative autistic traits and class membership.

For manuscripts utilizing custom algorithms or software that are central to the research but not yet described in published literature, software must be made available to editors and reviewers. We strongly encourage code deposition in a community repository (e.g. GitHub). See the Nature Portfolio guidelines for submitting code & software for further information.

Data

Policy information about availability of data

All manuscripts must include a data availability statement. This statement should provide the following information, where applicable:

- Accession codes, unique identifiers, or web links for publicly available datasets
- A description of any restrictions on data availability
- For clinical datasets or third party data, please ensure that the statement adheres to our policy

Due to data access and sharing restrictions, the data used in this study are not publicly available. For further information about data access, please contact the corresponding author.

Research involving human participants, their data, or biological material

Policy information about studies with human participants or human data. See also policy information about sex, gender (identity/presentation), and sexual orientation and race, ethnicity and racism.

Reporting on sex and gender	Sex was recorded and reported as sex assigned at birth: male or female
Reporting on race, ethnicity, or other socially relevant groupings	Ethnicity was comprised of 19 groups to select from, where the groups broadly came under the umbrellas 'White', 'Mixed', 'Asian', 'Black' and 'Other'. The full list of ethnicities can be found in the appendices.
Population characteristics	Age, sex assigned at birth, ethnicity, marital status, education level, employment status and volunteering activity were recorded from the full sample and included in the dataset. Age was recorded based on the age the participant was at the time of completing the Autism questionnaires. There was no data available for gender identity, so sex assigned at birth (male/female) was used. Marital status comprised of 7 options: 'Married', 'Widowed', 'Separated', 'Divorced', 'Civil Partnership', 'Co-habitating' and 'Single'. Education level was recorded as the highest education level that had been reached at the time of completing the Autism questionnaires. Participants selected from: 'Secondary Education', 'Post-Secondary Education', 'Vocational Qualification', 'Undergraduate Degree', 'Post-Graduate Degree' and 'Doctorate'. For simplicity of reporting, these were then grouped as 'School to 16 (secondary education)', 'School to 18 (post-secondary education & vocational qualification)','Undergraduate' and 'Postgraduate (including doctorate)'. Employment status was recorded as participant's current employment status at the time of completing the Autism questionnaire. Participants selected from 'Employed full-time', 'Employed part-time', 'Self-employed', 'Retired' and 'Unemployed' which were then condensed into three categories: 'Employed', 'Retired' and 'Unemployed'.
Recruitment	Participants were part of the wider PROTECT study (www.protectstudy.org.uk) which is a UK-wide research study that was launched in 2015 aimed at understanding brain changes and the underlying causes of dementia in middle and later adulthood (Corbett et al., 2023). Participants completed annual questionnaires centred around lifestyle, health and cognition. The initial PROTECT sample consisted of 20,200 adults aged over 50 years. Participants were recruited to the study via advertising from Alzheimer's Society UK, the UK Medical Research Council, press coverage by the British Broadcasting Corporation (BBC), and on social media. Inclusion criteria for the PROTECT study were adults aged over 50 years, resident in the United Kingdom, with a good understanding of English, and able to use a computer with internet access. Participants who had an established diagnosis of dementia at baseline were excluded. Eligible participants registered online and were required to read an information sheet before providing consent to take part. No additional recruitment took place for the purpose of the present study as the relevant questionnaires were already embedded into the existing PROTECT dataset. Further details of the PROTECT study can be found at http://www.protectstudy.org.uk.
Ethics oversight	The PROTECT study received ethical approval from the U.K. London Bridge National Research Ethics Committee (Ref: 13/LO/1578).

Note that full information on the approval of the study protocol must also be provided in the manuscript.

Field-specific reporting

Please select the one below that is the best fit for your research. If you are not sure, read the appropriate sections before making your selection.

☒ Life sciences ☐ Behavioural & social sciences ☐ Ecological, evolutionary & environmental sciences

For a reference copy of the document with all sections, see nature.com/documents/nr-reporting-summary-flat.pdf

Life sciences study design

All studies must disclose on these points even when the disclosure is negative.

Sample size	The sample size used in this study (n=5,270) across several timepoints is sufficiently powered for conducting GMM analyses. The sample size of each subgroup identified as been considered during the analysis phase and in the write-up of the manuscript.
Data exclusions	All participants (i.e., n=5,270) were included in the estimation to maximise sample size. Participants who had total GAD-7 scores for fewer

Data exclusions	than three timepoints (i.e., baseline plus two annual follow-up entries) were excluded from GMM analysis, as this is the recommended minimum requirement for proper estimation of GMMs.36 Full information maximum likelihood was used to handle missing data on GAD-7 scores.
Replication	Replication was not undertaken as part of this study.
Randomization	Randomisation did not take place for this study. Participants were assigned high and low AST groups based on their scores on the AST questionnaire.
Blinding	No blinding was used for this study.

Reporting for specific materials, systems and methods

We require information from authors about some types of materials, experimental systems and methods used in many studies. Here, indicate whether each material, system or method listed is relevant to your study. If you are not sure if a list item applies to your research, read the appropriate section before selecting a response.

Materials & experimental systems

n/a	Involved in the study
☒	☐ Antibodies
☒	☐ Eukaryotic cell lines
☒	☐ Palaeontology and archaeology
☒	☐ Animals and other organisms
☒	☐ Clinical data
☒	☐ Dual use research of concern
☒	☐ Plants

Methods

n/a	Involved in the study
☒	☐ ChIP-seq
☒	☐ Flow cytometry
☒	☐ MRI-based neuroimaging

Plants

Seed stocks	*Report on the source of all seed stocks or other plant material used. If applicable, state the seed stock centre and catalogue number. If plant specimens were collected from the field, describe the collection location, date and sampling procedures.*
Novel plant genotypes	*Describe the methods by which all novel plant genotypes were produced. This includes those generated by transgenic approaches, gene editing, chemical/radiation-based mutagenesis and hybridization. For transgenic lines, describe the transformation method, the number of independent lines analyzed and the generation upon which experiments were performed. For gene-edited lines, describe the editor used, the endogenous sequence targeted for editing, the targeting guide RNA sequence (if applicable) and how the editor was applied.*
Authentication	*Describe any authentication procedures for each seed stock used or novel genotype generated. Describe any experiments used to assess the effect of a mutation and, where applicable, how potential secondary effects (e.g. second site T-DNA insertions, mosiacism, off-target gene editing) were examined.*
